# Supplementary material for: SORBS2 is a genetic factor contributing to cardiac malformation of 4q deletion syndrome patients
Source: eLife. 2021 Jun 8;10:e67481. doi: 10.7554/eLife.67481 (PMC8186900; doi:10.7554/eLife.67481)
Supplement: Supplementary file 3. [file elife-67481-supp3.docx]

**Supplementary file 3. Genotyping distribution in embryos from *Sorbs2^+/-^* mouse intercross**

| **Embryonic stage** | **Total** | **Genotype of embryos** | | | **ASD** |
| --- | --- | --- | --- | --- | --- |
|  |  | **WT** | ***Sorbs2^+/-^*** | ***Sorbs2^-/-^*** |  |
| E18.5 | 137 | 42 | 65 | 30^a^ | 12^b^ |

a：The observed ratio is not different from the expected. Two-sided χ2 test (χ2=1.212, *p*=0.546).

b: 10 cases of primary septum hypoplasia/aplasia and 2 cases of double atrial septum.
