# Supplementary material for: SORBS2 is a genetic factor contributing to cardiac malformation of 4q deletion syndrome patients
Source: eLife. 2021 Jun 8;10:e67481. doi: 10.7554/eLife.67481 (PMC8186900; doi:10.7554/eLife.67481)
Supplement: Supplementary file 6. [file elife-67481-supp6.docx]

**Supplementary file 6. Number of exonic variants detected in CHD and normal controls.**

|  | **CHD cases** | **Normal controls** |
| --- | --- | --- |
| **All genes** |  |  |
| Total | 1108 | 842 |
| Synonymous SNV | 506 | 430 |
| Stopgains | 4 | 2 |
| Nonsynonymous SNV | 598 | 410 |
| **CHD genes** |  |  |
| Total | 899 | 658 |
| Synonymous SNV | 409 | 329 |
| Stopgains | 3 | 2 |
| Nonsynonymous SNV | 487 | 327 |
| **Candidate genes** |  |  |
| Total | 209 | 184 |
| Synonymous SNV | 97 | 101 |
| Stopgains | 1 | 0 |
| Nonsynonymous SNV | 111 | 83 |
