# Supplementary material for: SORBS2 is a genetic factor contributing to cardiac malformation of 4q deletion syndrome patients
Source: eLife. 2021 Jun 8;10:e67481. doi: 10.7554/eLife.67481 (PMC8186900; doi:10.7554/eLife.67481)
Supplement: Supplementary file 7. [file elife-67481-supp7.docx]

**Supplementary file 7. Carriers of rare damaging variants in CHD and normal controls.**

|  | **Gene** | **No.carriers in CHD patients (n=298)** | **No.carriers in controls (n=220)** | **p.value^*^** | **Odds Ratio** | **BH q.value^&^** |
| --- | --- | --- | --- | --- | --- | --- |
| 1 | *SORBS2* | 20 | 4 | 0.006129 | 3.876383 | 0.1920615 |
| 2 | *KMT2D* | 22 | 5 | 0.006739 | 3.420478 | 0.1920615 |
| 3 | *EVC2* | 13 | 3 | 0.04131 | 3.292905 | 0.6029175 |
| 4 | *SH3PXD2B* | 15 | 4 | 0.04231 | 2.857021 | 0.6029175 |
| 5 | *CHD7* | 7 | 1 | 0.08121 | 5.255293 | 0.925794 |
| 6 | *EHMT1* | 7 | 2 | 0.1859 | 2.617671 | 1 |
| 7 | *PTPN11* | 3 | 0 | 0.1896 | Inf | 1 |
| 8 | *ROR2* | 17 | 8 | 0.1907 | 1.601803 | 1 |
| 9 | *JAG1* | 5 | 1 | 0.1953 | 3.729429 | 1 |
| 10 | *EVC* | 10 | 4 | 0.2162 | 1.872866 | 1 |
| 11 | *FBN1* | 11 | 5 | 0.2558 | 1.646571 | 1 |
| 12 | *ACVR1* | 2 | 0 | 0.3305 | Inf | 1 |
| 13 | *PDLIM3* | 2 | 0 | 0.3305 | Inf | 1 |
| 14 | *SALL4* | 2 | 0 | 0.3305 | Inf | 1 |
| 15 | *TBX1* | 2 | 0 | 0.3305 | Inf | 1 |
| 16 | *NFATC1* | 17 | 10 | 0.3527 | 1.269886 | 1 |
| 17 | *COL2A1* | 8 | 4 | 0.368 | 1.488552 | 1 |
| 18 | *CREBBP* | 8 | 4 | 0.368 | 1.488552 | 1 |
| 19 | *ANKRD1* | 3 | 1 | 0.4325 | 2.223996 | 1 |
| 20 | *CALR* | 3 | 1 | 0.4325 | 2.223996 | 1 |
| 21 | *NODAL* | 3 | 1 | 0.4325 | 2.223996 | 1 |
| 22 | *STRA6* | 3 | 1 | 0.4325 | 2.223996 | 1 |
| 23 | *ZEB2* | 3 | 1 | 0.4325 | 2.223996 | 1 |
| 24 | *LBR* | 4 | 2 | 0.4929 | 1.481908 | 1 |
| 25 | *RAI1* | 13 | 9 | 0.5315 | 1.069274 | 1 |
| 26 | *SMAD6* | 2 | 1 | 0.6123 | 1.478654 | 1 |
| 27 | *TAB2* | 2 | 1 | 0.6123 | 1.478654 | 1 |
| 28 | *TBX3* | 2 | 1 | 0.6123 | 1.478654 | 1 |
| 29 | *KCNH2* | 3 | 2 | 0.6396 | 1.108287 | 1 |
| 30 | *JAK2* | 5 | 4 | 0.6811 | 0.9216485 | 1 |
| 31 | *NOTCH1* | 6 | 5 | 0.6981 | 0.8837761 | 1 |
| 32 | *NSD1* | 6 | 5 | 0.6981 | 0.8837761 | 1 |
| 33 | *ELN* | 7 | 6 | 0.7134 | 0.8582199 | 1 |
| 34 | *MYH6* | 15 | 13 | 0.7378 | 0.844262 | 1 |
| 35 | *ALDH1A2* | 4 | 4 | 0.7875 | 0.7351475 | 1 |
| 36 | *NF1* | 4 | 4 | 0.7875 | 0.7351475 | 1 |
| 37 | *TBX5* | 2 | 2 | 0.7921 | 0.7369374 | 1 |
| 38 | *PTCH1* | 7 | 7 | 0.8035 | 0.7324166 | 1 |
| 39 | *FOXH1* | 1 | 1 | 0.8201 | 0.7378233 | 1 |
| 40 | *LEFTY2* | 1 | 1 | 0.8201 | 0.7378233 | 1 |
| 41 | *RAF1* | 1 | 1 | 0.8201 | 0.7378233 | 1 |
| 42 | *TBX20* | 1 | 1 | 0.8201 | 0.7378233 | 1 |
| 43 | *NOS3* | 5 | 6 | 0.8697 | 0.6092539 | 1 |
| 44 | *MED13L* | 7 | 8 | 0.8701 | 0.6380351 | 1 |
| 45 | *MYH7* | 3 | 4 | 0.8794 | 0.549811 | 1 |
| 46 | *VEGFA* | 3 | 4 | 0.8794 | 0.549811 | 1 |
| 47 | *PDGFRA* | 2 | 3 | 0.8937 | 0.4894172 | 1 |
| 48 | *NPHP3* | 4 | 7 | 0.9589 | 0.4147024 | 1 |
| 49 | *GATA4* | 1 | 3 | 0.968 | 0.2441802 | 1 |
| 50 | *ZFPM2* | 2 | 5 | 0.9747 | 0.2912188 | 1 |
| 51 | *MYH11* | 0 | 6 | 1 | 0 | 1 |
| 52 | *NKX2-6* | 0 | 3 | 1 | 0 | 1 |
| 53 | *DLL1* | 0 | 2 | 1 | 0 | 1 |
| 54 | *EFNB2* | 0 | 2 | 1 | 0 | 1 |
| 55 | *F7* | 0 | 2 | 1 | 0 | 1 |
| 56 | *NOTCH2* | 0 | 2 | 1 | 0 | 1 |
| 57 | *SLC2A10* | 0 | 2 | 1 | 0 | 1 |

*p.values are calculated with one-tailed Fisher’s exact test

&q.values are adjusted p-values after Benjamini-Hochberg testing.
