# Supplementary material for: SORBS2 is a genetic factor contributing to cardiac malformation of 4q deletion syndrome patients
Source: eLife. 2021 Jun 8;10:e67481. doi: 10.7554/eLife.67481 (PMC8186900; doi:10.7554/eLife.67481)
Supplement: Supplementary file 9. [file elife-67481-supp9.docx]

**Supplementary file 9. Cardiac phenotype in patients carrying *SORBS2* variants.**

| **Subjects** | **Cardiac Phenotypes*** |
| --- | --- |
| P1 | TOF ASD PDA |
| P2 | PA VSD SV |
| P3 | DORV VSD PH |
| P4 | DORV CAVC VSD ASD PH |
| P5 | TOF ASD |
| P6 | TOF ASD PDA |
| P7 | DORV VSD PH |
| P8 | SA CAVC Dextrocardia PA |
| P9 | PA VSD ASD Dextrocardia |
| P10 | TOF ASD |
| P11 | TOF ASD |
| P12 | DORV VSD PH ASD |
| P13 | TA VSD ASD PH |
| P14 | TGA VSD PH |
| P15 | TGA VSD PDA ASD |
| P16 | TGA VSD ASD |
| P17 | DORV VSD ASD PH |
| P18 | TOF ASD |
| P19 | SV PA ASD |
| P20 | PA VSD |

*: TOF: Tetralogy of Fallot; ASD: Atrial septal defect; PDA: Patent ductus arteriosus; PA: Pulmonary atresia; VSD: Ventricular septal defect; SV: Single ventricle; DORV: Double outlet right ventricle; PH: Pulmonary hypertension; CAVC: complete atrioventricular septal defect; SA: Single atrium; TA: Tricuspid atresia; TGA: Transposition of great arteries.
