# Supplementary material for: SORBS2 is a genetic factor contributing to cardiac malformation of 4q deletion syndrome patients
Source: eLife. 2021 Jun 8;10:e67481. doi: 10.7554/eLife.67481 (PMC8186900; doi:10.7554/eLife.67481)
Supplement: Supplementary file 10. [file elife-67481-supp10.docx]

**Supplementary file 10. Primers for qPCR.**

| **Gene** | **Organism** | **Forward 5’- 3’** | **Reverse 5’- 3’** |
| --- | --- | --- | --- |
| *OCT4* | Human | GGGGTTCTATTTGGGAAGGT | CTGGTTCGCTTTCTCTTTCG |
| *NANOG* | Human | CAGCCCCGATTCTTCCACCAGTCCC | CGGAAGATTCCCAGTCGGGTTCACC |
| *SOX2* | Human | ATGACCAGCTCGCAGACCTA | GACTTGACCACCGAACCCAT |
| *T* | Human | TATGAGCCTCGAATCCACATAGT | GTTCCTCCATCATCTCTTTGTGA |
| *MESP1* | Human | TCGAAGTGGTTCCTTGGCAGAC | CCTCCTGCTTGCCTCAAAGTGTC |
| *TBX5* | Human | AGACTCGCTGCTGAAAGGAC | GGAGCTGCACAGAATGTCAA |
| *HAND1* | Human | AATCCTCTTCTCGACTGGGC | CCTTCAAGGCTGAACTCAAGA |
| *HCN4* | Human | GACACCGCTATCAAAGTGGA | AGGTCCCAGTAAAATCTGAAGT |
| *ISL1* | Human | TCACGAAGTCGTTCTTGCTG | CATGCTTTGTTAGGGATGGG |
| *MEF2C* | Human | TTTCCTGTTTCCTCCAAACAA | CCAAGGACTAATCTGATCGGG |
| *TBX1* | Human | CGGCTCCTACGACTATTGCCC | GGAACGTATTCCTTGCTTGCCCT |
| *NRARP* | Human | CCAACTGCGAGTTCAACGTG | GGGAAGGTACAGCAGAGACG |
| *HEY1* | Human / Mus | TGGTACCCAGTGCTTTTGAG | CTCCGATAGTCCATAGCAAGG |
| *HEYL* | Human / Mus | ATGCAAGCCAGGAAGAAACGCAGA | AGCTTGGAAGAGCCCTGTTTCTCA |
| *PTCH1* | Human | CCACAGAAGCGCTCCTACA | CTGTAATTTCGCCCCTTCC |
| *GLI1* | Human | CTCCCGAAGGACAGGTATGTAAC | CCCTACTCTTTAGGCACTAGAGTT |
| *cTnT* | Human | ATGAGCGGGAGAAGGAGCGGCAGAAC | TCAATGGCCAGCACCTTCCTCCTCTC |
| *MYH7* | Human | ACCAACCTGTCCAAGTTCCG | TTCAAGCCCTTCGTGCCAAT |
| *MYH6* | Human | TCCGTGAAGGGATAACCAGG | ACAGTCACCGTCTTCCCATTC |
| *MLC-2A* | Human | TCAAAGAAGCCTTCAGCTGTATC | TGAACTCATCCTTGTTCACCAC |
| *BMP4* | Human | CGATGTGGGCTGGAATGA | TGGTTGAGTTGAGGTGGTCAG |
| *SMAD3* | Human | CATCGAGCCCCAGAGCAATA | GTGGTTCATCTGGTGGTCACT |
| *ID2* | Human | TGGACTCGCATCCCACTATT | CAGAAGCCTGCAAGGACAG |
| *MSX1* | Human | CTCGTCAAAGCCGAGAGC | CGGTTCGTCTTGTGTTTGC |
| *FGF8* | Human | TGAGCTGCCTGCTGTTGCACTT | TGAAGACGCAGTCCTTGCCTT |
| *FGF18* | Human | GGACATGTGCAGGCTGGGCTA | GTAGAATTCCGTCTCCTTGCCCTT |
| *FZD5* | Human | CTTGTTTCCAAAGTCCAATCAAGTG | GCCTACTCTTCACCCTTCTTTAACG |
| *FOXF1* | Human | CAGCCGTATCTGCACCAGAA | ACTCCTTTCGGTCACACATGCT |
| *PTCH1* | Mus | GGACCGTGTCCTGAGGTGTCT | GGCAAACCGGACGACACTT |
| *GLI1* | Mus | TTATGGAGCAGCCAGAGAGA | GAGCCCGCTTCTTTGTTAAT |
