# Supplementary material for: SORBS2 is a genetic factor contributing to cardiac malformation of 4q deletion syndrome patients
Source: eLife. 2021 Jun 8;10:e67481. doi: 10.7554/eLife.67481 (PMC8186900; doi:10.7554/eLife.67481)
Supplement: Supplementary file 11. [file elife-67481-supp11.docx]

**Supplementary file 11. Subphenotypes of CHD cohort.**

| **Cardiac Phenotypes** | **Subjects** | **Subjects Frequency**  **(%)** | | |
| --- | --- | --- | --- | --- |
| Tetralogy of Fallot | 89 (46/43)^*^ | | 29.7 |  |
| Pulmonary atresia | 60 (19/41)^*^ | | 20 |  |
| Double outlet right ventricle | 59 (30/29)^*^ | | 19.7 |  |
| Transposition of great arteries | 46 (29/17)^*^ | | 15.3 |  |
| Single atrium/single ventricle | 27 | | 9 |  |
| Tricuspid atresia | 11 | | 3.7 |  |
| Interrupted aortic arch | 8 | | 2.7 |  |
| **Total** | 300 | |  |  |

*, numbers within parentheses indicate the numbers of patients with/without ASD.
