# Supplementary material for: SORBS2 is a genetic factor contributing to cardiac malformation of 4q deletion syndrome patients
Source: eLife. 2021 Jun 8;10:e67481. doi: 10.7554/eLife.67481 (PMC8186900; doi:10.7554/eLife.67481)
Supplement: Supplementary file 12. [file elife-67481-supp12.docx]

**Supplementary file 12. Known CHD genes included in targeted sequencing panel.**

| **Gene** | **Phenotype** | **OMIM** | **Cytogenetic location** | **Genomic location(GRCh38)** |
| --- | --- | --- | --- | --- |
| *ACTC1* | ASD/VSD | 102540 | 15q14 | chr15: 34,788,095-34,795,725 |
| *ACVR1* | BAV/AVSD | 102576 | 2q24.1 | chr2: 157,736,445-157,875,895 |
| *ACVR2B* | Heterotaxy/PS/DORC/TGA | 602730 | 3p22.2 | chr3: 38,454,298-38,493,141 |
| *ALDH1A2* | TOF | 603687 | 15q21.3 | chr15: 57,953,423-58,065,922 |
| *ANKRD1* | TAPVC | 609599 | 10q23.31 | chr10: 90,912,099-90,921,274 |
| *ATRX* | Turner syndrome | 300032 | Xq21.1 | chrX: 77,504,877-77,786,268 |
| *BCOR* | Heterotaxy | 300485 | Xp11.4 | chrX: 40,051,245-40,177,389 |
| *BRAF* | Noonan/Cardiofaciocutaneous syndrome | 164757 | 7q34 | chr7: 140,719,326-140,924,927 |
| *CFC1* | Heterotaxy/TOF/TGA/IAA | 605194 | 2q21.1 | chr2: 130,592,164-130,599,574 |
| *CHD7* | Charge syndrome | 608892 | 8q12.2 | chr8: 60,678,743-60,868,027 |
| *CITED2* | ASD/VSD | 602937 | 6q24.1 | chr6: 139,372,254-139,374,649 |
| *COL2A1* | Stickler syndrome/AVSD | 120140 | 12q13.11 | chr12: 47,972,964-48,006,211 |
| *CREBBP* | Rubinstein-Taybi syndrome/ ventricular septation | 600140 | 16p13.3 | chr16: 3,725,053-3,880,726 |
| *CRELD1* | AVSD/ASD | 607170 | 3p25.3 | chr3: 9,933,787-9,945,412 |
| *CSDE1* | 1p13.2 microdeletion | 191510 | 1p13.2 | chr1: 114,716,912-114,758,049 |
| *EHMT1* | Kleefstra syndrome | 607001 | 9q34.3 | chr9: 137,618,991-137,836,126 |
| *ELN* | PPS/SVAS/AS | 130160 | 7q11.23 | chr7: 74,027,771-74,069,906 |
| *EVC* | Ellis-van Creveld syndrome | 604831 | 4p16.2 | chr4: 5,711,196-5,829,042 |
| *EVC2* | Ellis-van Creveld syndrome | 607261 | 4p16.2 | chr4: 5,562,407-5,709,547 |
| *FBN1* | BAV/Marfan syndrome | 134797 | 15q21.1 | chr15: 48,408,305-48,645,787 |
| *FLNA* | ASD | 300017 | Xq28 | chrX: 154,348,531-154,374,637 |
| *FOXC1* | TOF/Aortic valve dysplasia | 601090 | 6p25.3 | chr6: 1,610,445-1,613,896 |
| *FOXH1* | TOF/TGA/ASD/IAA | 603621 | 8q24.3 | chr8: 144,473,731-144,476,334 |
| *FOXL2* | VSD | 605597 | 3q22.3 | chr3: 138,944,223-138,947,139 |
| *G6PC3* | ASD/PVS/PDA | 611045 | 17q21.31 | chr17: 44,070,699-44,076,343 |
| *GATA4* | ASD/AVSD/TOF/VSD | 600576 | 8p23.1 | chr8: 11,676,918-11,760,001 |
| *GATA6* | ASD/PTA/PS/TOF | 601656 | 18q11.2 | chr18: 22,169,436-22,202,527 |
| *GDF1* | DORV/Heterotaxy/TGA/TOF | 602880 | 19p13.11 | chr19: 18,868,545-18,896,143 |
| *GJA1* | AVSD/HLHS | 121014 | 6q22.31 | chr6: 121,435,576-121,449,743 |
| *GPC3* | DORV | 300037 | Xq26.2 | chrX: 133,535,744-133,985,645 |
| *HAND2* | VSD/PS/TOF | 602407 | 4q34.1 | chr4: 173,526,500-173,530,226 |
| *HEY2* | TOF | 604674 | 6q22.31 | chr6: 125,747,638-125,762,242 |
| *HOXA1* | VSD | 142955 | 7p15.2 | chr7: 27,092,992-27,096,005 |
| *HRAS* | Costello syndrome/ASD/VSD | 190020 | 11p15.5 | chr11: 532,241-535,566 |
| *IRX4* | VSD | 606199 | 5p15.33 | chr5: 1,877,426-1,887,178 |
| *JAG1* | Alagille syndrome/TOF | 601920 | 20p12.2 | chr20: 10,637,683-10,674,045 |
| *KMT2D* | HLHS/VSD/ASD | 602113 | 12q13.12 | chr12: 49,018,974-49,060,883 |
| *KRAS* | Noonan/Cardiofaciocutaneous syndrome | 190070 | 12p12.1 | chr12: 25,204,788-25,251,002 |
| *LBR* | Human CHD Gene | 600024 | 1q42.12 | chr1: 225,401,501-225,428,854 |
| *LEFTY2* | Heterotaxy/TGA/AVSD | 601877 | 1q42.12 | chr1: 225,936,597-225,941,491 |
| *MAP2K1* | Cardiofaciocutaneous syndrome | 176872 | 15q22.31 | chr15:66,386,872-66,491,543 |
| *MAP2K2* | Cardiofaciocutaneous syndrome | 601263 | 19p13.3 | chr19:4,090,320-4,124,183 |
| *MED13L* | TGA | 608771 | 12q24.21 | chr12: 115,958,575-116,277,218 |
| *MGP* | Keutel syndrome | 154870 | 12p12.3 | chr12: 14,880,863-14,886,061 |
| *MID1* | Opitz syndrome | 300552 | Xp22.2 | chrX: 10,445,309-10,833,689 |
| *MYH11* | PDA/TAAD | 160745 | 16p13.11 | chr16: 15,703,134-15,857,031 |
| *MYH6* | ASD/Tricuspid atresia | 160710 | 14q11.2 | chr14: 23,381,989-23,408,276 |
| *MYH7* | Ebstein’s anomaly of tricuspid valve/ASD | 160760 | 14q11.2 | chr14: 23,412,737-23,435,685 |
| *NF1* | TOF | 613113 | 17q11.2 | chr17: 31,094,926-31,377,676 |
| *NKX2-5* | ASD/HLHS/TOF/VSD | 600584 | 5q35.1 | chr5: 173,232,103-173,235,320 |
| *NKX2-6* | PTA/VSD/TOF/DORV | 611770 | 8p21.2 | chr8: 23,702,450-23,706,597 |
| *NODAL* | Heterotaxy/TOF | 601265 | 10q22.1 | chr10: 70,431,935-70,447,947 |
| *NOTCH1* | BAV/LVOTO/TOF | 190198 | 9q34.3 | chr9: 136,494,432-136,545,785 |
| *NOTCH2* | Alagille syndrome | 600275 | 1p12 | chr1: 119,911,552-120,069,702 |
| *NPHP3* | ASD/PTA/PDA | 608002 | 3q22.1 | chr3: 132,680,608-132,722,458 |
| *NRAS* | Noonan syndrome | 164790 | 1p13.2 | chr1: 114,704,463-114,716,893 |
| *NSD1* | Sotos syndrome | 606681 | 5q35.3 | chr5: 177,131,834-177,300,212 |
| *PDGFRA* | TAPVC | 173490 | 4q12 | chr4: 54,229,088-54,298,246 |
| *PTPN11* | Noonan/LEOPARD syndrome | 176876 | 12q24.13 | chr12: 112,418,897-112,509,917 |
| *RAF1* | Noonan/LEOPARD syndrome | 164760 | 3p25.2 | chr3: 12,583,600-12,664,200 |
| *RAI1* | Smith-Magenis syndrome | 607642 | 17p11.2 | chr17: 17,681,375-17,811,452 |
| *RBM10* | TARP syndrome | 300080 | Xp11.3 | chrX: 47,145,195-47,186,814 |
| *ROR2* | Robinow syndrome | 602337 | 9q22.31 | chr9: 91,722,595-91,950,205 |
| *SALL4* | Duane-radial ray syndrome/VSD | 607343 | 20q13.2 | chr20: 51,782,716-51,802,522 |
| *SH3PXD2B* | Frank-Ter Haar syndrome | 613293 | 5q35.1 | chr5: 172,325,180-172,454,522 |
| *SHOC2* | Noonan syndrome | 602775 | 10q25.2 | chr10: 110,919,369-111,013,666 |
| *SLC2A10* | Arterial tortuosity syndrome | 606145 | 20q13.12 | chr20: 46,708,357-46,736,346 |
| *SMAD6* | BAV/AS/COA | 602931 | 15q22.31 | chr15: 66,702,109-66,781,999 |
| *SOS1* | Noonan syndrome | 182530 | 2p22.1 | chr2: 38,981,548-39,124,958 |
| *STRA6* | PDAC syndrome | 610745 | 15q24.1 | chr15: 74,179,465-74,212,266 |
| *TAB2* | VSD/BAV/LVOTO | 605101 | 6q25.1 | chr6: 149,217,923-149,411,612 |
| *TBX1* | DiGeorge syndrome/TOF | 602054 | 22q11.21 | chr22: 19,756,702-19,783,592 |
| *TBX20* | TOF/PTA/ASD/VSD | 606061 | 7p14.2 | chr7: 35,199,935-35,254,099 |
| *TBX3* | Ulnar-mammary syndrome | 601621 | 12q24.21 | chr12: 114,670,253-114,684,163 |
| *TBX5* | Holt-Oram syndrome | 601620 | 12q24.21 | chr12: 114,353,910-114,408,707 |
| *TDGF1* | TOF/VSD | 187395 | 3p21.31 | chr3: 46,574,554-46,582,462 |
| *TFAP2B* | PDA/PTA/TOF | 601601 | 6p12.3 | chr6: 50,817,691-50,847,618 |
| *VEGFA* | TOF | 192240 | 6p21.1 | chr6: 43,770,208-43,786,486 |
| *ZEB2* | Mowat-Wilson syndrome | 605802 | 2q22.3 | chr2: 144,384,374-144,520,390 |
| *ZFPM2* | TOF | 603693 | 8q23.1 | chr8: 105,318,858-105,804,538 |
| *ZIC3* | Heterotaxy/TGA/ASD/PS | 300265 | Xq26.3 | chrX: 137,566,126-137,577,690 |
