# Supplementary material for: SORBS2 is a genetic factor contributing to cardiac malformation of 4q deletion syndrome patients
Source: eLife. 2021 Jun 8;10:e67481. doi: 10.7554/eLife.67481 (PMC8186900; doi:10.7554/eLife.67481)
Supplement: Supplementary file 13. [file elife-67481-supp13.docx]

| **Supplementary file 13. Candidate CHD genes included in targeted sequencing panel.** | | | |
| --- | --- | --- | --- |
| **Gene** | **OMIM** | **Cytogenetic location** | **Genomic location(GRCh38)** |
| *CALR* | 109091 | 19p13.13 | chr19: 12,938,599-12,944,489 |
| *CNN1* | 600806 | 19p13.2 | chr19: 11,538,716-11,550,322 |
| *CRK* | 164762 | 17p13.3 | chr17: 1,421,352-1,456,266 |
| *DLL1* | 606582 | 6q27 | chr6: 170,282,199-170,290,608 |
| *EFNB2* | 600527 | 13q33.3 | chr13: 106,489,730-106,535,039 |
| *EPOR* | 133171 | 19p13.2 | chr19: 11,377,204-11,384,341 |
| *ETS1* | 164720 | 11q24.3 | chr11: 128,458,760-128,587,592 |
| *F7* | 613878 | 13q34 | chr13: 113,105,772-113,120,680 |
| *FLI1* | 193067 | 11q24.3 | chr11: 128,685,262-128,813,266 |
| *GJA5* | 121013 | 1q21.2 | chr1: 147,756,198-147,781,126 |
| *HEY1* | 602953 | 8q21.13 | chr8: 79,764,010-79,767,863 |
| *JAK2* | 147796 | 9p24.1 | chr9: 4,985,085-5,128,182 |
| *KCNH2* | 152427 | 7q36.1 | chr7: 150,944,955-150,978,313 |
| *MYCN* | 164840 | 2p24.3 | chr2: 15,940,437-15,947,006 |
| *NFATC1* | 600489 | 18q23 | chr18: 79,395,771-79,529,322 |
| *NOS3* | 163729 | 7q36.1 | chr7: 150,991,055-151,014,598 |
| *PDLIM3* | 605889 | 4q35.1 | chr4: 185,500,659-185,535,557 |
| *PTCH1* | 601309 | 9q22.32 | chr9: 95,442,979-95,517,056 |
| *QKI* | 609590 | 6q26 | chr6: 163,414,485-163,578,595 |
| *RPS6KA2* | 601685 | 6q27 | chr6: 166,409,363-166,862,550 |
| *SHH* | 600725 | 7q36.3 | chr7: 155,799,983-155,812,272 |
| *SLC25A4* | 103220 | 4q35.1 | chr4: 185,143,262-185,150,383 |
| *SORBS2* | 616349 | 4q35.1 | chr4: 185,585,443-185,956,715 |
